# Econobiophysics - game of choosing. Model of selection or election process with diverse accessible information

**DOI:** 10.1186/1753-4631-5-7

**Published:** 2011-09-05

**Authors:** Wlodzimierz Klonowski, Michal Pierzchalski, Pawel Stepien, Robert A Stepien

**Affiliations:** 1 Nalecz Institute of Biocybernetics and Biomedical Engineering, Polish Academy of Sciences, 4 Trojdena St., 02-109 Warsaw, Poland

**Keywords:** econophysics,  preferences,  impact,  selection process, mating, monogamic, polygamic,  election  campaign,  spin,  influence of environment

## Abstract

We propose several models applicable to both *selection *and *election processes *when each selecting or electing subject has access to different information about the objects to choose from. We wrote special software to simulate these processes. We consider both the cases when the environment is neutral (natural process) as well as when the environment is involved (controlled process).

## Introduction

We consider a system **U** consisting of two classes of elements - a class **S** of *subjects *that are *active*, that is they can make decisions to choose (to select or to elect) elements they somehow prefer from a class **O **of *objects*. Objects are *passive *i.e. they can only compete to be chosen (selected or elected). Subjects and objects are embedded in a much greater space called *environment*, **E** (cf. [1]).

Let us assume that the class **S** consists of *N *subjects A, B, C, ... and the class of objects **O** consists of *M *subclasses α, β, γ, ... with *i_m _*objects belonging to the *m*th subclass (m = 1, ... , *j_m_*) - *j_1 _*objects a belonging to subclass α, *j_2 _*objects b belonging to subclass β, etc. In the simplest case each subclass consists of only one object.

Subjects' preferences are given in the form of *preference matrix P = [p_mn_] *where the element *p_mn _*denotes preference of the *n*-th subject towards *m*-th subclass of objects.

We call the process in the considered system **U** the *Game of Choosing, GoC*. GoC is characterized by discrete time, *t*****, that counts number of rounds of the GoC from the initial moment. Each round means *exposition *of the objects to the subjects. The periods between the subsequent rounds may differ from one another. Exposition may be direct (one may call it *sensorial*) presentation of the object to the subject or it may be just a transmission of information about the object to the subjects through the environment, for example presentation of a TV spot. For a subject to win GoC means to choose the most preferred object before other subjects would do this or to choose more objects than other subjects during the same period of time. For an object to win GoC means to be chosen before other objects would be.

Subjects and objects exchange information through the environment. The environment may be *neutral*, i.e. it does not influence transmitted information; in such a case we call the process *natural GoC *as for example when *choosing a mating partner*. The environment may be *active*, i.e. it can somehow transform information it transmits; in such a case we call it *controlled GoC *as for example when *choosing a product *based on information added by Google every time one searches the web or uses Gmail or when *electing an MP *influenced by public relation (PR) propaganda.

For simplicity we will assume that each subclass consists of only one object, so there are *M *objects and *N *active subjects. Subjects' preferences are equal to probability of choosing given object multiplied by 100. If *p_mn _*= 50 it means '50-50' situation, that is equal probability of choosing and of rejecting object *m *by subject *n*. If *p_mn _*= *p_t _*= 80 it means that probability of choosing the object *m *by subject *n *is four-times greater than probability of rejecting it.

**Figure 1:**
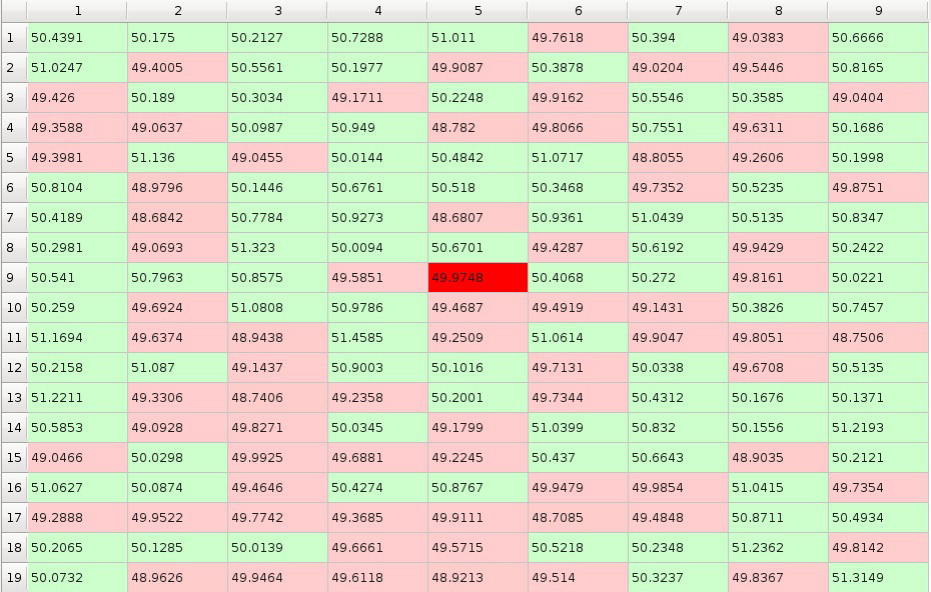
**Example of preference matrix**.

**Figure 2:**
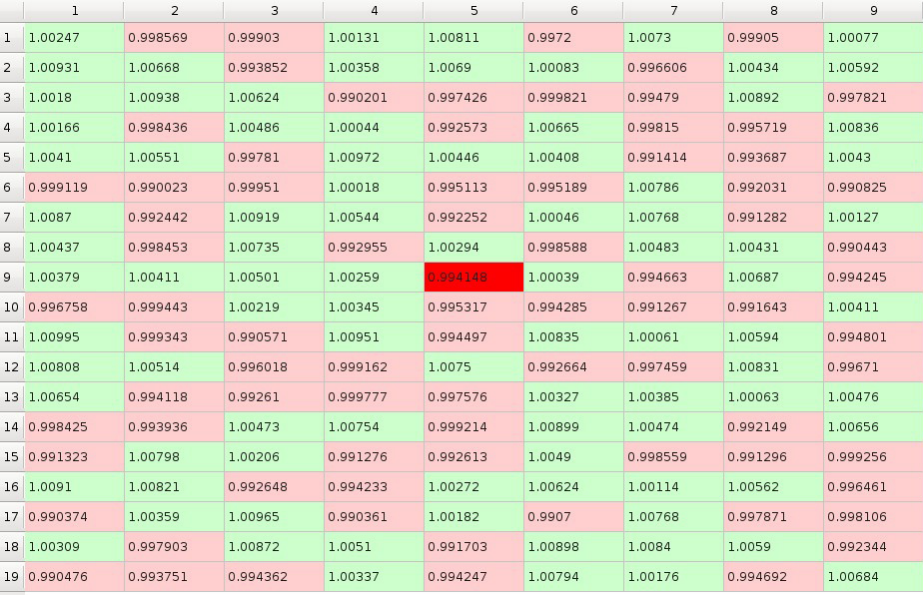
**Example of impact matrix**.

In the beginning we initialize preference matrix *P *with values close to 50, between 48.5 and 51.5, i.e. all subjects have very similar preferences to all objects but some preferences are slightly greater than others (Figure 1). We assume that the influence of the objects on the subjects is expressed in the form of the *impact matrix I = [i_mn_] *where the element *i_mn _*denotes influence of one exposition of the *m*-th object on the preference of the *n*-th subject towards this object (Figure 2). And the evolution of the system in time is given by

(1) $$p_{mn}\left(t+1\right)=p_{mn}{\left(t\right)}_{*}i_{mn}\left(t\right)$$

by multiplying element by element (not multiplication of the matrices!), where *t *denotes the number of rounds of GoC. If *i_mn_(t) = 1 *it means that the exposition does not change the preference of the *n*-th subject towards the *m*-th object, what is equivalent to the assertion that the *m*-th *o*bject was not at all exposed to the *n*-th subject in the round *t*. It makes possible to adopt a simplifying assumption that all objects are exposed to all subjects during each round of GoC.

If *i_mn_(t) > 1 *it means that the exposition number *t *causes increase of the preference of the *n*-th subject towards the *m*-th object; if *i_mn_(t) < 1 *the exposition causes decrease of the preference. So, the state of the system after *t *rounds of GoC depends on subjects' initial preferences as well as on the impact matrix and its evolution in time.

We consider several variants of GoC with different interactions between subjects, objects, and environment. Our models may be applied both in biomedical systems as well as in socio-economical systems. By analogy to *econophysics *we will call it *econobiophysics*.

In the matrices columns correspond to subjects and rows correspond to objects, and the 'compartment' at the crossing of column *n *with the row *m *that may be filled up with a value of the corresponding element will be called the *cell*. Preference matrix cell is of green shade if the preference is higher than 50 and otherwise it is of red shade. Impact matrix cell is of green shade if the impact is higher than 1.0 and otherwise it is of red shade. So, in the figures and in the moving pictures (cf. additional files) green shade signifies that the *m*-th object is being chosen by the *n*-th subject and red shade that it is being rejected. We generate starting *p_mn _*values to be close to 50 - between 48.5 and 51.5 and *i_mn _*values to be close to 1 - between 0.99 and 1.01. The game starts from a random point which is marked with more intensive colour then others. In the following example (Figure 1 and Figure 2) the game starts from the 9-th row and the 5-th column.

To each model below the corresponding short moving picture showing the course of GoC is added in an additional files that for convenience we will call 'Picture Q.' where Q is the serial number of the Figure to which it corresponds (so there are no Picture 1. nor Picture 2. between the additional files). Each moving picture illustrates dynamics of the GoC, presented as changing of cells' colours with time. The end of each game is shown in the corresponding figure and when a number pops up in a cell it shows how many rounds of GoC have been necessary for the subject *n *to make decision to accept (green colour) or to reject (red colour) the object *m*. The colour of the cells and its intensity changes during the course of the game, with increasing intensities corresponding to increasing probabilities of acceptation or rejection - the greater is the intensity of colour the closer is the subject to final decision of choosing or rejecting the object. Intensity of colour changes instantly if the subject makes the decision. For convenience, only the 'winning/loosing' numbers are displayed at the end of the game showing how many rounds (expositions) each subject needed before making the decision; these numbers are also shown in the corresponding figure...

## Results

### I. Natural GoC - 'monogamic model'

**Figure 3:**
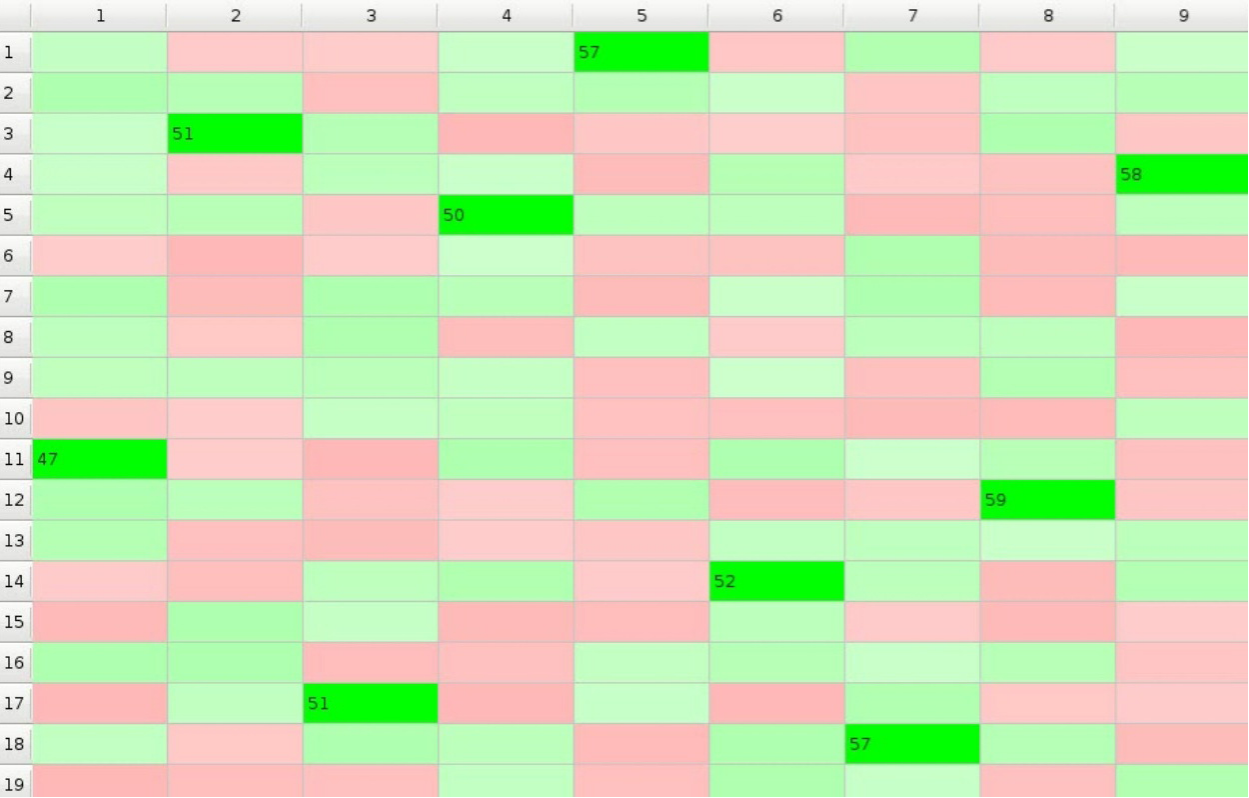
**GoC - monogamic model**.

In the simplest model we assume that the environment is neutral and the impact matrix *I *does not change with time; in such a case the results of selection process depend only on the number of times the objects reveal their impact on the subjects. At the moment when some preference *p_mn _*exceeds the threshold *p_t_*******both the subject *n *and the object *m *stop to take part in the GoC while the game may continues until either all subjects would make their choice or until all available objects would be chosen. Natural selection 'monogamic model' is illustrated in Additional File 1. Picture 3. that results with Figure 3.

In this example subject 1. chose object number 11. after 47 rounds, while subject 8. needed 59 rounds to choose object 12. 10 out of 19 subjects lost the game i.e. they were not chosen at all.

### II. Natural GoC - 'polygamic model'

**Figure 4:**
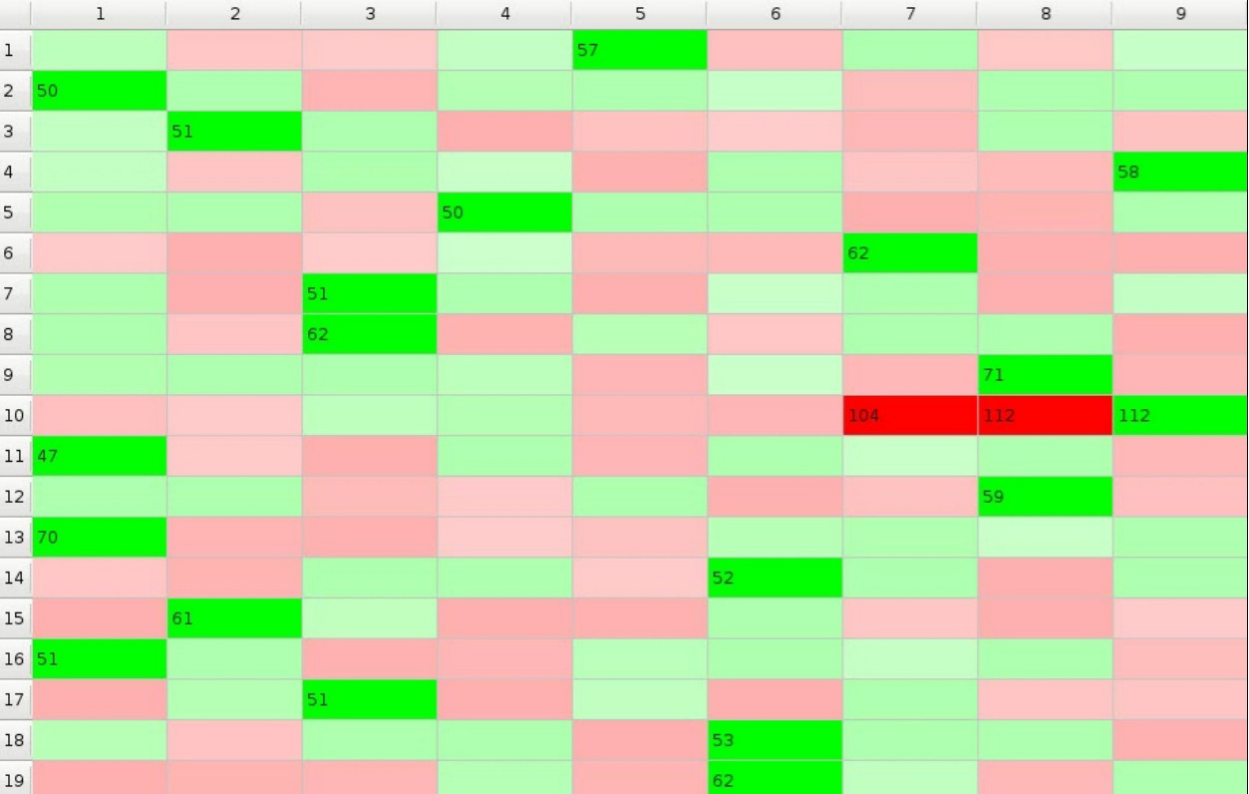
**GoC - polygamic model**.

The difference between the 'polygamic model' and the 'monogamic model' lies in the assumption that at the moment when some preference *p_mn _*exceeds the threshold *p_t _*only ****the object *m *stops to take part in the GoC while the subject *n *remains in the game and GoC continues. So, each subject may choose more than one object and at the end of the game all objects are chosen. Natural selection 'polygamic model' is illustrated in Additional File 2. Picture 4. that results with Figure 4. Object 10. is not being chosen for quite a long period to be finally rejected by subjects 7. and 8., but at the same moment it is chosen by subject 9. as the last free object of all, so the game is over - subjects 4., 5., and 7. have only one object each, while subject 1. has as many as four objects.

### III. Natural GoC with interaction between subjects

In this model subjects do not act independently. The values of preference matrix for subject *n*****change depending on the preferences of the nearest neighbours subjects (cf. [2]) and the evolution of the system in time is given by Eq. (2) instead of Eq. (1):

(2) $$p\left(m,n\right)=\frac{\overset{a}{\underset{k=-a}{\sum }}\overset{b}{\underset{l=-b}{\sum }}i\left(m+k,n+l\right)*p\left(m+k,n+l\right)}{\overset{a}{\underset{k=-a}{\sum }}\overset{b}{\underset{l=-b}{\sum }}i\left(m+k,n+l\right)}$$

where *a *= (*g*-1)/2 and *b = *(*h*-1)/2; g_*_h is the size of mask defining the nearest neighbours. For convenience, in the above formula we used indices in parenthesis instead of lower indices, so *p(m, n) *means the element *p_mn _*of the preference matrix, *i(m, n) *- the element ***i***_***mn ***_of the impact matrix, and the multiplication in the numerator means multiplying element by element. As in Eq. (1), the left-hand side of Eq. (2) corresponds to the round *t+1 *of the GoC, while right-hand side to the preceding round *t*. In the following example the mask is 1 × 5. Toroidal 'boundary condition' are assumed for both preference and impact matrix so every subject and every object has two nearest neighbours.

**Figure 5:**
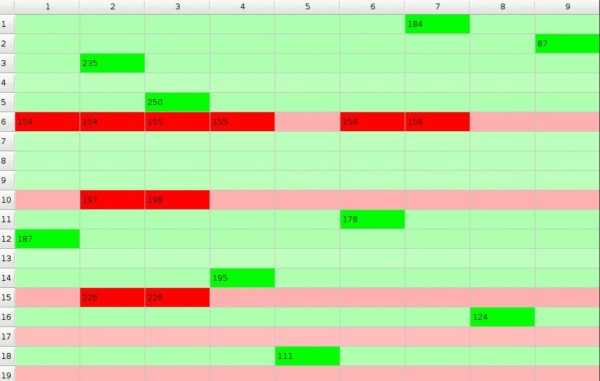
**Natural GoC with interaction between subjects - monogamic variant with one subclass**.

**Figure 6:**
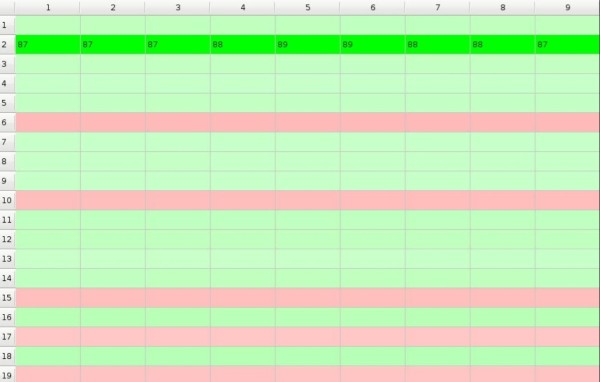
**Natural GoC with interaction between subjects - monogamic variant with the number subclass at least equal to the number of subjects**.

Each subject tries to be as similar to its neighbours as possible so the preferences values *p_mn_(t+1) *increase relatively to *p_mn_(t) *for all objects belonging to the subclass *j_m _*if preferences of the neighbouring subjects towards objects belonging to this subclass are greater than the preference *p_mn_(t) *of the considered *n*-th subject. Subjects make their choices depending not only on their own but also on their neighbours' preferences. In monogamic model (Figure 3.) subject 7. chose object number 18. In this case for model with one subclass (see Additional File 3. Picture 5., and Figure 5.) subject 7. chose objects 1. because of 'friendly neighbourhood'. Six out of nine subjects rejected object 6. For the model with the number of subclasses at least equal to the number of subjects every choice is rather choice of the whole group (see Additional File 4. Picture 6., and Figure 6.) The most popular object in this example is the object number 2. Each of the two models may also have a 'polygamic' variant.

Both models may easily be modified in such a way to simulate the case when each subject tries to be as different from its neighbours as possible. Mixed cases may be simulated as well.

### IV. Controlled GoC with feedback between subjects and the environment

In the previous models elements of the impact matrix ***i_mn _***were generated randomly at the beginning of the game and then remained unchanged. In the controlled GoC models impact matrix does change - if a subject *n *shows at the moment *t = 0 *preference *p_mn _*for an object *m *the environment 'puts pressure' on the subject to choose or to reject object *m *independently of the impact of the object itself. So, we introduce a generalization of the impact matrix so called *external impact matrix*, *e_mn_(t)*

(3) $$e_{mn}\left(t\right)=a_{*}p_{mn}\left(t\right)+b_{*}i_{mn}\left(t\right)+c_{mn}\left(t\right)+d_{*}\eta \left(t\right)$$

(4) $$p_{mn}\left(t+1\right)=p_{mn}{\left(t\right)}_{*}e_{mn}\left(t\right)$$

where *c_mn_(t) *denotes environmental pressure exerted on the subject *n *to choose (*c_mn_(t) > 0*) or to reject (*c_mn_(t) < 0*) object *m*; *η(t) *denote noise; *a*, *b*, and *d *are constants (cf. Eq. (1)); again, a star denotes multiplying element by element, not multiplication of the matrices. If *c_mn_(t) *is not equal zero it means that the environment exerts pressure e.g. by 'taking a notice' of subject's *n *preferences towards the object *m *and passing to the subject *n *information about the object *m *that is not normally presented by the object *m *itself. This way subject *n *becomes more and more encouraged or discouraged to choose object *m *and the impact of the object itself (expression b_*_i_mn_(t) in Eq. (3)) may often be much smaller than such an environmental pressure. Coefficient *a *in (3) measures 'stiffness' of the subjects' own preferences.

In the following examples we consider the simplest case - all *c_mn_(t) *are identical and remain constant, equal ***c ***; we also assume *b *= 0 and *d *= 0; for simplicity we also assume that each objects' subclass consists of only one object. For better comparison with natural GoC models discussed above in the following simulations we chose *a *= 0.001 and ***c ***= 0.950 to start with *e_mn_(0) *values from similar range as *i_mn_(t) *values in the previously discussed models (cf. Figure 2) while *p_mn_(0) *remains the same as previously (Figure 1).

**Figure 7:**
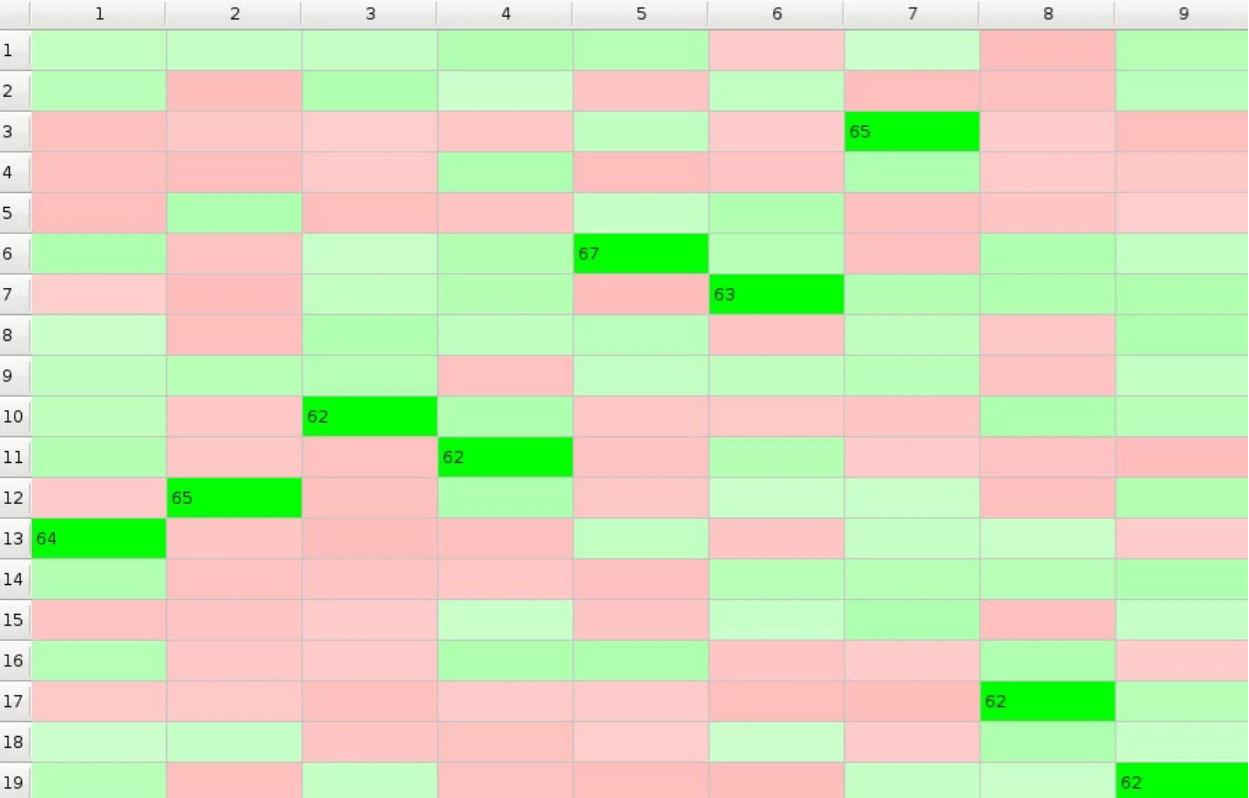
**Controlled GoC with feedback between subjects and the environment - monogamic variant**.

**Figure 8:**
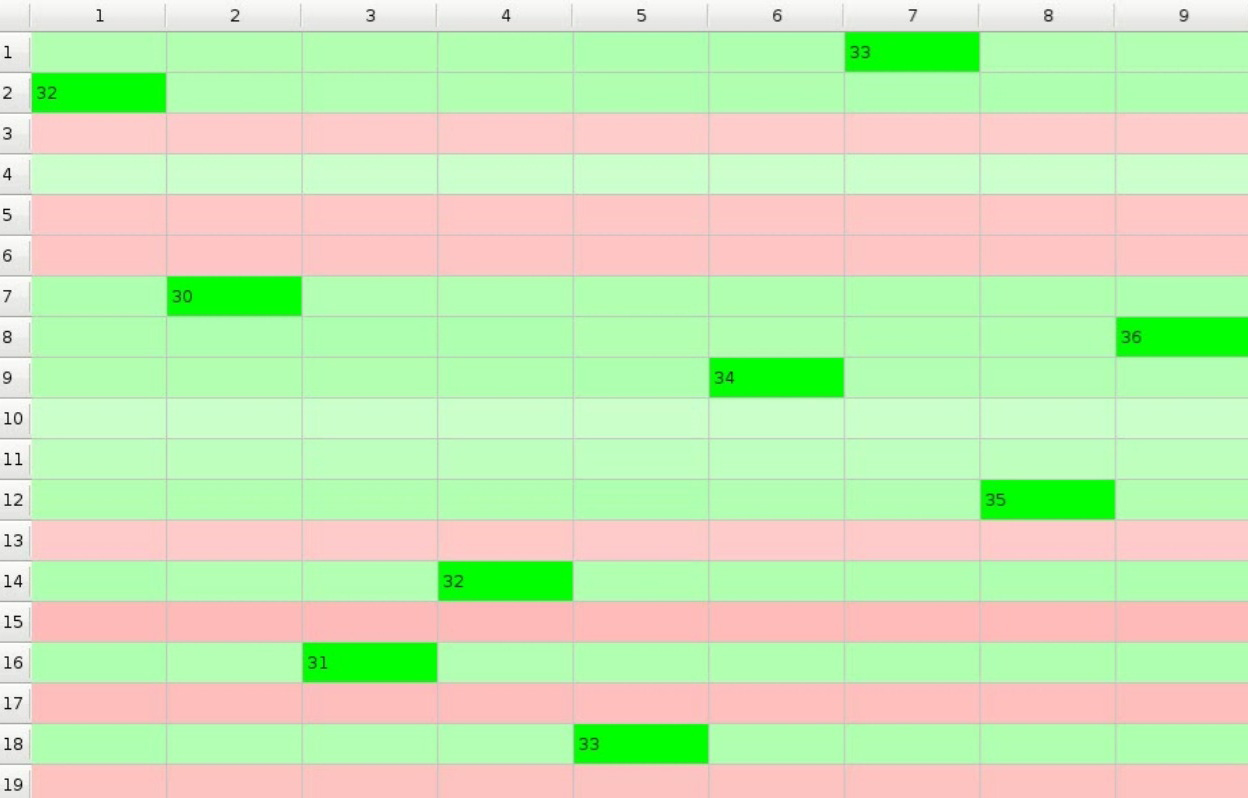
**Controlled GoC with feedback between subjects and the environment and with interactions with other subjects - monogamic variant**.

We consider here two models - controlled GoC with feedback between subjects and the environment, 'monogamic' variant, without interactions between the subjects (see Additional File 5. Picture 7., and Figure 7) and with additional interactions between subject as given by Eq. (2) (see Additional File 6. Picture 8., and Figure 8). In this last case in particular final preferences may be significantly different from the initial ones. This model may work for example with Google advertisement - if one has shown for example interest about China, Google with each searching or opening of Gmail will display information like 'Big discounts for flights to China', 'Thousands cheap hotels in China' etc. If somebody has shown interests for example about Brazil, Google will display similar kind of information about Brazil, etc. This model is also the very base of political propaganda, in particular during elections. Each of the two models may also have a 'polygamic' variant.

## Conclusions

In all above examples the subjects made their decisions of choosing while having only limited information about the objects they were choosing from. Moreover, each of the subjects had accessible different information, so each subject had different preferences.

Even the simplest 'monogamic model' clearly demonstrates how choosing for example a mating partner may just be a 'deterministic chaotic' game. Even miniscule differences in initial conditions (initial preferences) and/or small differences in number of times prospective partners are exposed to the subject while looking for a partner may cause very big differences in the final outcome i.e. in the mating pairs that would be finally formed. More complicated models show that influence of the environment may cause changes of initial preferences and so formation of very different mating pairs.

In commerce influence of the social environment is even more important. E.g. while choosing a model of a car one is deciding to buy the problem which car models the neighbours possess might be of primary importance in two quite opposite ways - one may want to be 'just like others' or one may want to differ as much as possible.

But the same models also well illustrate 'games' played during any election campaign and by governments, big companies, etc in general. Famous statement attributed to Joseph Goebbels, minister of propaganda of the III Reich, states: "If you tell a lie big enough and keep repeating it, people will eventually come to believe it...." However, most probably similar statement was even earlier formulated by Vladimir Lenin: "A lie told often enough becomes truth". These statement became a base of propaganda in all authoritarian regimes.

The father of modern Psychology William James (1842-1910) said: "There's nothing so absurd that if you repeat it often enough, people will believe it." And 'the Father of Spin' Edward L. Bernays, a nephew to Sigmund Freud, in 1920s created what is now called PR by observing how masses of people could be swayed through messages repeated over and over hundreds of times [3]. In science frequently undetected fraudulent data and results in peer-reviewed journals are quoted by other researchers, who are in turn re-quoted by still others, and so on ([4,5]).

'Regularity' that a lie if repeated hundreds times becomes the truth is similar to money savings with constant compound interests. So called 'rule 72' says that if one subdivides 72 by the percentage added per annum the result will be a good approximation of the period (expressed in years) necessary for the investment to double itself. Similarly, if one subdivides 72 by (*| i_mn _*- 1|_*_100) one calculates the approximate number of expositions necessary for the preference of the *n*-th subject towards the *m*-th object to increase twofold under assumption that the impact matrix *I *does not change with time. One should not forget 'negative impact' - if *i_mn_(t) < 1 *each exposition discourages subject *n*****from choosing object *m*. So, a minuscule difference in initial preferences or a miniscule difference in impact values may lead to dramatically different results of GoC even in the simplest cases.

So, *econobiophysics *does have a very broad spectrum of application.

## Competing interests

The authors declare that they have no competing interests.

## Authors' contributions

WK - main ideas and writing the paper. MP - main computer programming, graphics generation, model improvement. PS - programming and model improvement. RS - programming and model improvement. All authors read and approved the manuscript.

## Supplementary Material

Additional file 1 **Picture 3**. GoC - monogamic model. Click here for file

Additional file 2 **Picture 4**. GoC - polygamic model. Click here for file

Additional file 3 **Picture 5**. Natural GoC with interaction between subjects - monogamic variant with one subclass. Click here for file

Additional file 4 **Picture 6**. Natural GoC with interaction between subjects - monogamic variant with the number subclass at least equal to the number of subjects. Click here for file

Additional file 5 **Picture 7**. Controlled GoC with feedback between subjects and the environment - monogamic variant. Click here for file

Additional file 6 **Picture 8**. Controlled GoC with feedback between subjects and the environment and with interactions with other subjects - monogamic variant. Click here for file
